# A bidirectional fabric-based soft robotic glove for hand function assistance in patients with chronic stroke

**DOI:** 10.1186/s12984-023-01250-4

**Published:** 2023-09-21

**Authors:** Daniel Yuan-Lee Lim, Hwa-Sen Lai, Raye Chen-Hua Yeow

**Affiliations:** 1 Evolution Innovation Lab, Advanced Robotics Centre, National University of Singapore, Singapore, Singapore; 2 Department of Biomedical Engineering, National University of Singapore, Singapore, Singapore; 3 Computer Science & Artificial Intelligence Laboratory, Massachusetts Institute of Technology, Cambridge, USA

**Keywords:** Soft robotics, Chronic stroke, Activities of daily living, Assistive device, Fabric-based actuator, Case Series

## Abstract

**Background:**

Chronic stroke patients usually experience reduced hand functions, impeding their ability to perform activities of daily living (ADLs) independently. Additionally, improvements in hand functions by physical therapy beyond six months after the initial onset of stroke are much slower than in the earlier months. As such, chronic stroke patients could benefit from an assistive device to enhance their hand functions, allowing them to perform ADLs independently daily. In recent years, soft robotics has provided a novel approach to assistive devices for motor impaired individuals, offering more compliant and lightweight alternatives to traditional robotic devices. The scope of this study is to demonstrate the viability of a fabric-based soft robotic (SR) glove with bidirectional actuators in assisting chronic stroke study participants with hand impairments in performing ADLs.

**Methods:**

Force and torque measurement tests were conducted to characterize the SR Glove, and hand functional tasks were given to eight chronic stroke patients to assess the efficacy of the SR Glove as an assistive device. The tasks involved object manipulation tasks that simulate ADLs, and the series of tasks was done by the participants once without assistance for baseline data, and once while using the SR Glove. A usability questionnaire was also given to each participant after the tasks were done to gain insight into how the SR Glove impacts their confidence and reliance on support while performing ADLs.

**Results:**

The SR Glove improved the participants’ manipulation of objects in ADL tasks. The difference in mean scores between the unassisted and assisted conditions was significant across all participants. Additionally, the usability questionnaire showed the participants felt more confident and less reliant on support while using the SR Glove to perform ADLs than without the SR Glove.

**Conclusions:**

The results from this study demonstrated that the SR Glove is a viable option to assist hand function in chronic stroke patients who suffer from hand motor impairments.

**Supplementary Information:**

The online version contains supplementary material available at 10.1186/s12984-023-01250-4.

## Background

Stroke is the most common cerebrovascular disease [1] and while the majority of stroke patients survive, up to 30% of all stroke survivors experience limited motor function recovery [2]. Consequently, stroke is also one of the main causes of adult disability [3], and many victims experience a reduction of hand motor function, restricting the victim’s ability to perform activities of daily living (ADLs) independently, negatively impacting their quality of life. Stroke patients with hand impairments require physical therapy, usually involving repetitive task practice rehabilitation [4], to improve hand function in terms of the range of motion and strength. However, around 65% of stroke patients are still unable to incorporate their affected hand into ADLs six months after the initial onset of stroke [5] and only 25% of patients return to a functional level similar to that of community-matched persons who have not suffered from a stroke [6]. In many of these chronic stroke cases, the patients enroll in step-down care services, where they undergo regular physical therapy. However, the rate of recovery of upper extremity function beyond six months after stroke is a lot slower than during the first three to six months [7]. Therefore, there is a need for assistive devices to aid chronic stroke patients in enhancing their hand functions to perform ADLs, regain independence in their daily lives and to enhance their recovery beyond six months after the initial onset of stroke.

Robotics has been presented as a non-invasive method of improving hand motor function using wearable actuators such as robotic exoskeletons. These exoskeletons aid the users in moving their fingers and hands. While several robotic exoskeletons have been developed for the upper limb, many rely on linear actuators and rigid linkages [8–10] aligned precisely to the users’ joints to transfer forces safely and efficiently. The rigid mechanical design of these exoskeletons limits portability due to their size and weight. As a result, many of these systems are stationary, designed solely for use in clinical settings and to be operated by professionals to ensure the patient’s safety. On top of that, their rigid nature leads to a reduction in patient comfort when using the exoskeletons. These conditions are not ideal to be used regularly as assistive devices.

Lightweight, user-friendly, and portable exoskeletons that could be used in both clinical and home settings as assistive devices would help create new possibilities that would be more suited for chronic stroke patients in day-to-day life. In recent years, several wearable hand exoskeletons have been developed using soft robotics. These compliant wearable exoskeletons incorporate fabrics [11, 12], polymers [13] and soft elastomeric actuators [14–16] instead of traditional rigid linkages, which allow for a more comfortable experience for the patients, as well as eliminate the need for complicated mechanical setups and trained personnel to operate. These novel approaches to wearable robotics introduce the possibility for safe and user-friendly devices for daily assistance at home or therapy over prolonged periods [14–17].

The objective of this study is to investigate the viability of a fully fabric-based soft robotic (SR) glove with bidirectional (i.e., finger flexion and finger extension) actuators in assisting chronic stroke study participants with hand mobility impairments in performing ADLs. To assess the effectiveness of the SR Glove as an assistive device, the study team adapted a series of hand functional tasks based on recognized and widely used hand assessments for stroke patients. The user-friendly design of the SR Glove module allowed participants to independently control the SR Glove using the Robotic Glove Control Box with minimal training. In addition to assessing the mechanical assistance by the glove, each participant did a usability questionnaire to gather information about how using the SR Glove affects their confidence in performing the ADL tasks as well as their reliance on support for performing ADLs with and without the SR Glove.

## Methods

### SR glove module

**Fig. 1:**
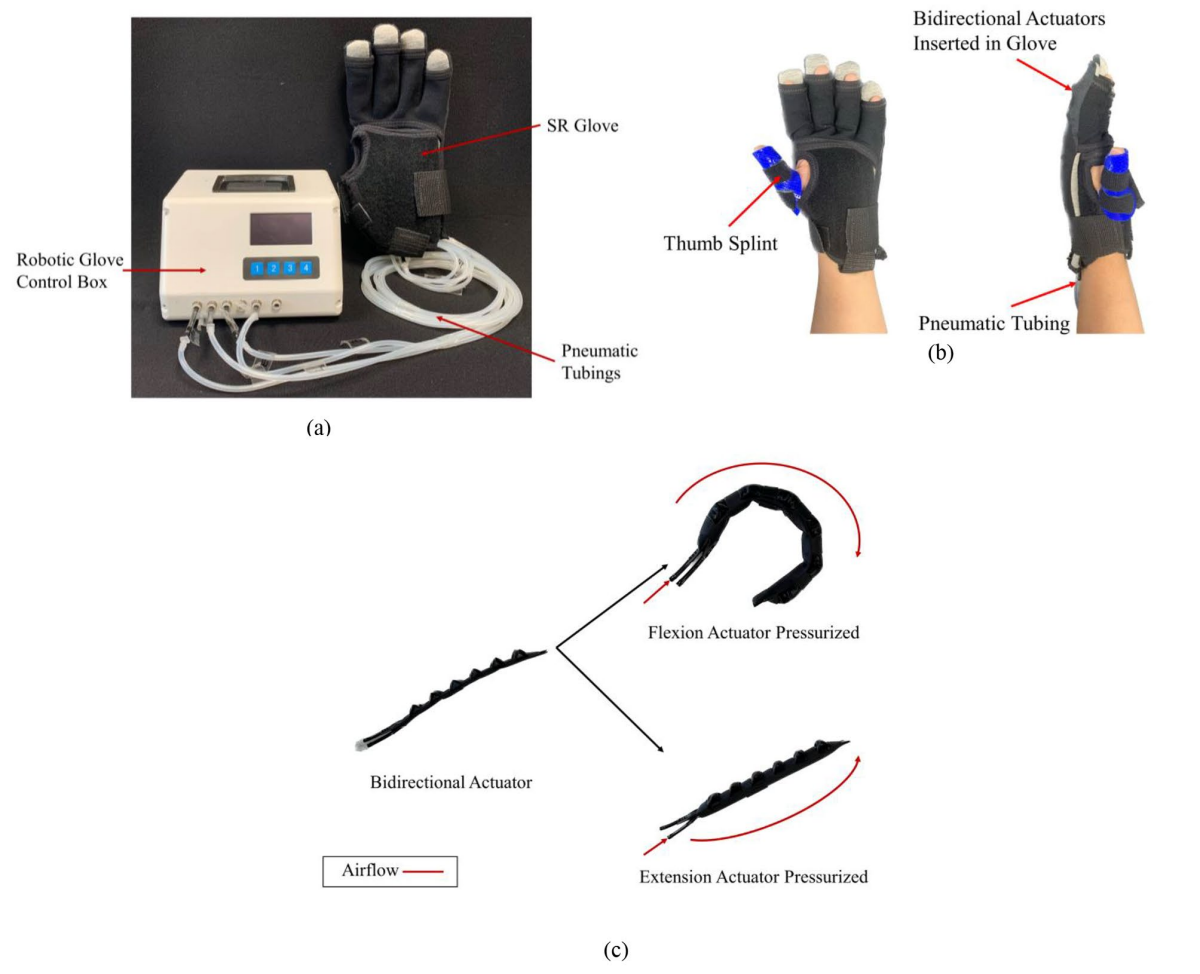
SR Glove module: **(a)** The SR Glove Module, consisting of the SR Glove and the Robotic Glove Control Box. **(b)** The SR Glove worn on a left hand, showing the thermoset thumb splint and pneumatic tubing from the actuators inserted into the dorsal side of the glove. **(c)** Fabric bidirectional actuators inserted in the dorsal side of the fabric glove and aligned with the four fingers. Each actuator has two modes of movement (flexion and extension) and is the basis of how the SR Glove assists the users in finger flexion and extension respectively

The SR Glove module [11] used in this study comprises of two parts: the SR Glove and the Robotic Glove Control Box (Fig. 1a). The Robotic Glove Control Box controls the activation of the actuators embedded in the SR Glove which in turn dictates what movements the SR Glove will assist the patient with.

The SR Glove consists of the fabric glove and four pneumatic actuators that are inserted in the dorsal side of the fabric glove, aligned with the four fingers (excluding the thumb). To address the lack of fine motor control and misalignment of most stroke survivor’s thumbs, participants wore a thumb splint made of thermoset plastic to assist in holding the thumb in opposition with the four fingers (Fig. 1b). This was necessary to allow the users to perform the tasks in the trial, such as grasping objects.

The actuators (Fig. 1c) in the SR Glove are bidirectional actuators that are fabricated entirely out of fabric. Each bidirectional actuator is made of two separate pneumatic actuators, the flexion actuator, and the extension actuator, which are secured together with thermoplastic polyurethane (TPU) fabric. At the base of both actuators, pneumatic tube adapters are inserted to serve as air inlets. The extension actuator resembles a traditional flat fabric pneumatic actuator. The flexion actuator, while similar to the extension actuator, is folded in a corrugated manner. The design of the corrugated fold allows the flexion actuator to achieve a bending motion, mimicking normal finger flexion when it is pressurised. Before pressurisation, both actuators are compliant and flexible. This compliance in their non-pressurised state is essential in ensuring that the range of motion of the participant’s fingers is not impeded. Compared to a previous iteration [11], the actuators presented in this paper are fabricated with a stiffer material (500GSM Double TPU Coated 420D Nylon).

**Fig. 2:**
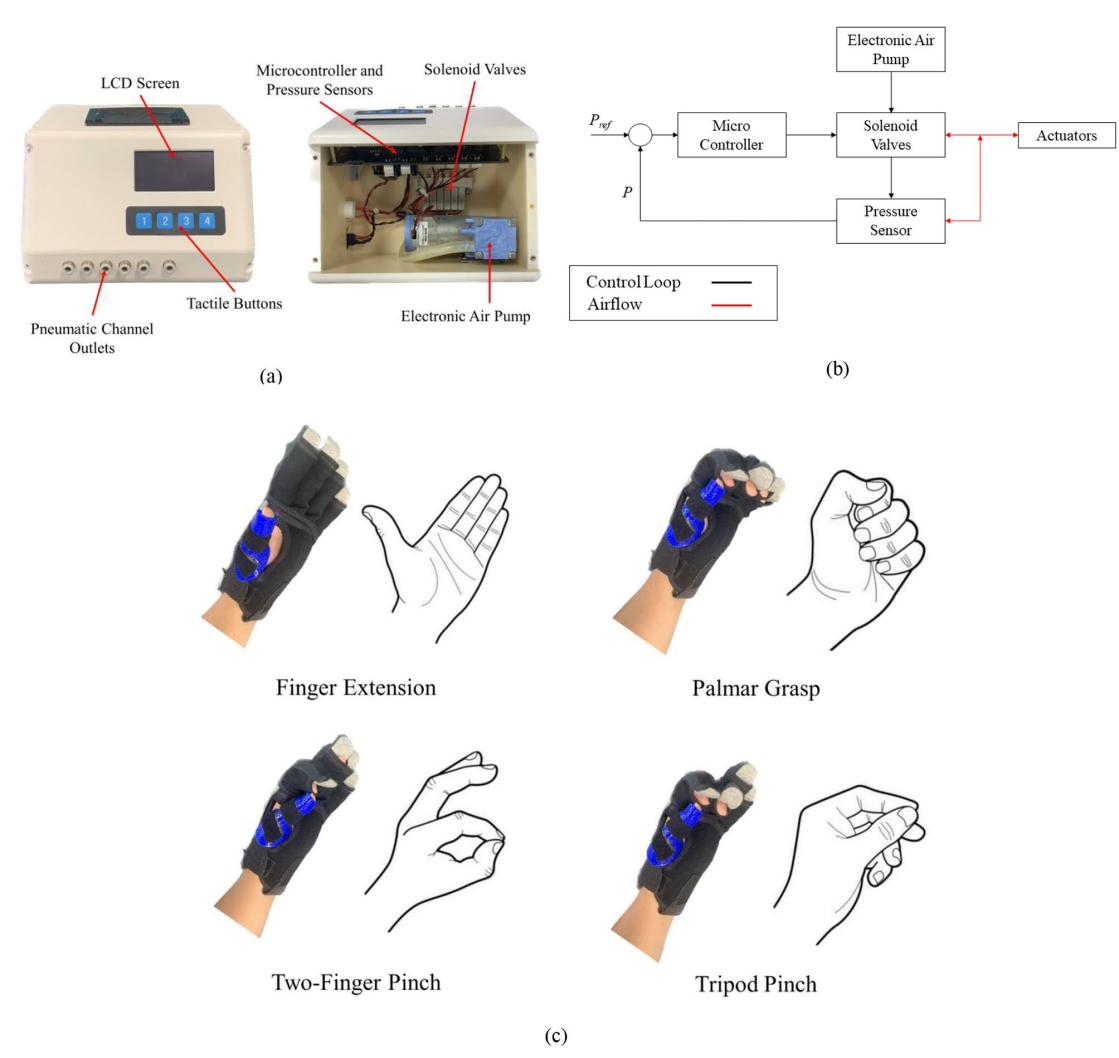
SR Glove module: **(a)** The Robotic Glove Control Box and its internals. A pressure control loop was implemented to drive the valves upon selection of the desired movement with the tactile buttons. **(b)** Control block diagram depicting the pressure control loop implemented in the Robotic Glove Control Box. **(c)** Control and coordination of the valves within the Robotic Glove Control Box help the SR Glove assist the user in four finger extension, palmar grasp, two-finger pinch, three-finger tripod pinch

The Robotic Glove Control Box (Fig. 2a) comprises a microcontroller, an electronic air pump, pressure sensors and solenoid valves. A pressure control loop (Fig. 2b) was implemented to drive the valves. Each valve is connected to an actuator in the SR Glove via pneumatic tubing. The control box also includes buttons to select the desired movement for the exercise and an LCD screen to visually assist the user in selecting the movements. The default available movements were Palmar Grasp, 2-point Pinch (index finger flexion), 3-point Tripod Pinch (index and middle finger flexion) and Extend (all four fingers extension) (Fig. 2c). As a movement is selected, the microcontroller activates the air compressor and opens the valves leading to the actuators that are meant to be activated, resulting in the desired movement of the SR Glove.

### Actuator characterization and force measurements

#### Flexion actuators

The flexion actuators were characterised and evaluated in terms of the blocked tip force and grip forces applied upon pressurisation.

**Fig. 3:**
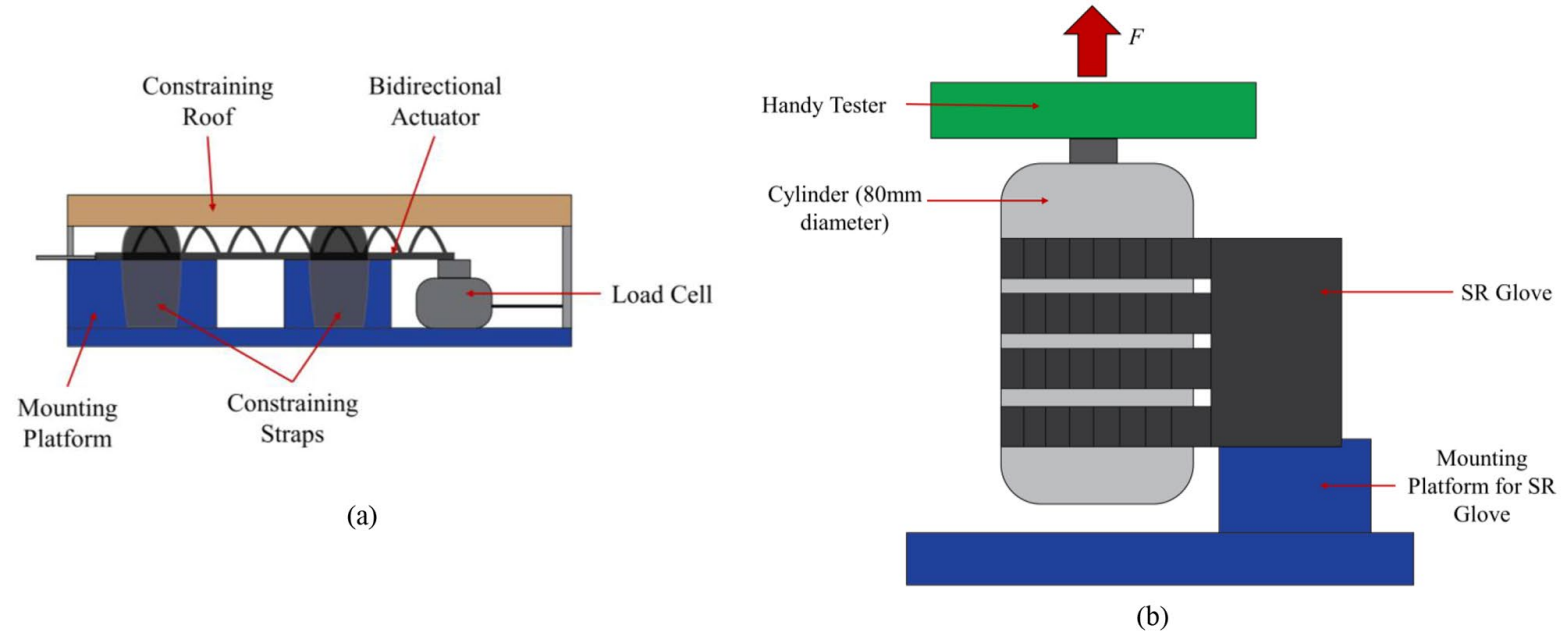
**(a)** Test setup for Blocked Tip Force measurements. The bidirectional actuator is held in place by a constraining roof and constraining straps to minimise non-linear effects caused by the bending of the flexion actuator when pressurised and force produced by the actuator is measured by the Load Cell. **(b)** Test setup for Frictional Grip Force measurements. The flexion actuators are pressurized to 130 kPa and grip onto the cylinder mounted on the handy tester universal testing machine in a vertical orientation. The cylinder is pulled upwards at a fixed velocity of 8 mm/s to the point the actuators released the cylinder

Blocked tip force measurements were done using a customised force measurement setup, which comprised a mounting platform for the flexion actuator and a compression load cell (Fig. 3a). The force measurement setup was similar to that of previous studies [16, 18, 19]. When pressurized, the flexion actuator’s bending motion is constrained by the two adjustable straps, which minimises the non-linear effects caused by the bending of the actuator when pressurised. This in turn helps ensure that the compression load cell would read the maximum blocked tip force generated by the actuator regardless of the bend angle. The distal end of the actuator was in contact with the compression load cell. Force was measured at pressure intervals of 10 kPa from 10 kPa to 130 kPa and repeated three times at every interval.

**Fig. 4:**
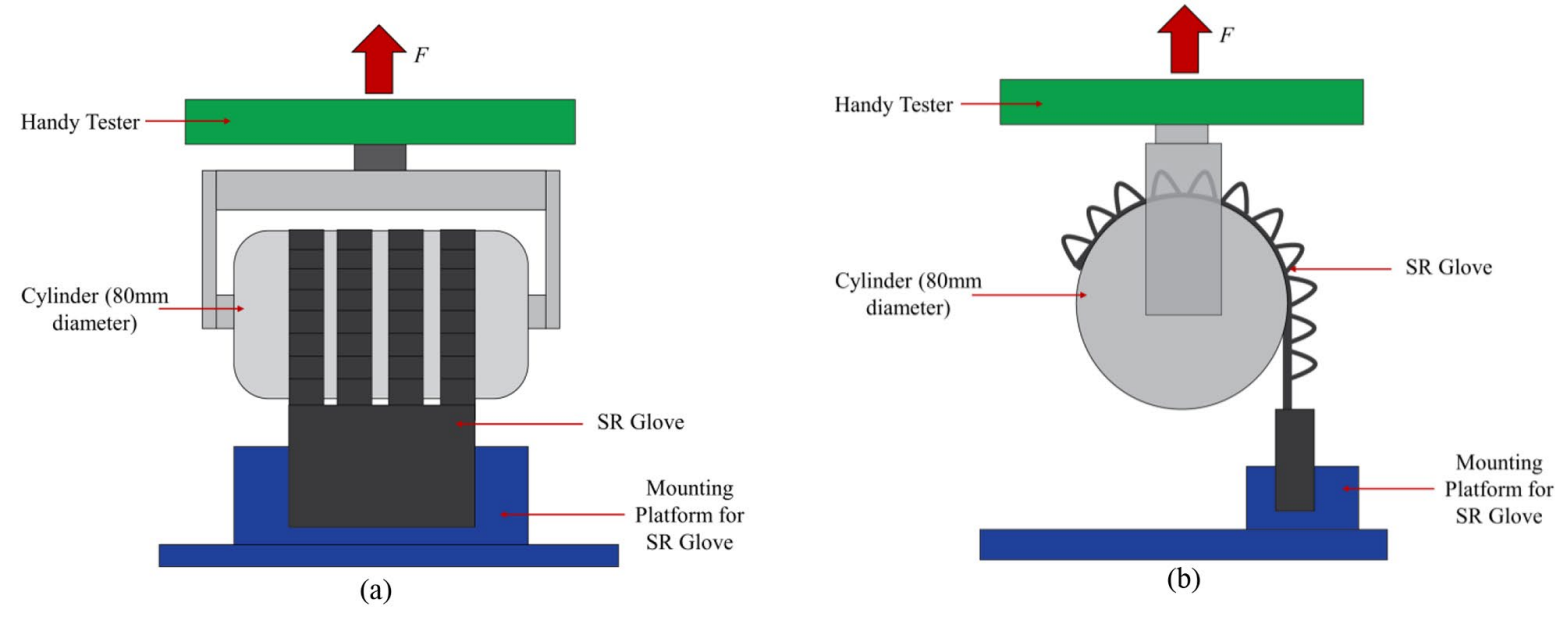
Two different angles of the test setup for Normal Grip Force measurements, **(a)**: front view, **(b)**: side view. The flexion actuators are pressurized to 130 kPa and grip onto the cylinder mounted on the handy tester universal testing machine in a horizontal orientation. The cylinder is pulled upwards at a fixed velocity of 8 mm/s to the point the actuators released the cylinder

The grip force applied by the flexion actuators was measured with a universal testing machine (Handy Tester JSV H10000) to obtain normal and frictional grip forces. The force measurement setup was similar to those reported in previous literature [16, 18, 20]. Four actuators, corresponding to the four fingers, were pressurised to 130 kPa to bend and grasp a cylinder that is 80 mm in diameter in a vertical orientation for frictional grip force (Fig. 3b), and horizontal orientation for normal grip force (Fig. 4). The cylinder was then pulled upwards by the universal testing machine at a fixed velocity of 8 mm/s to the point where the actuators release the cylinder. The tests were repeated three times and the results were averaged.

#### Extension actuators

**Fig. 5:**
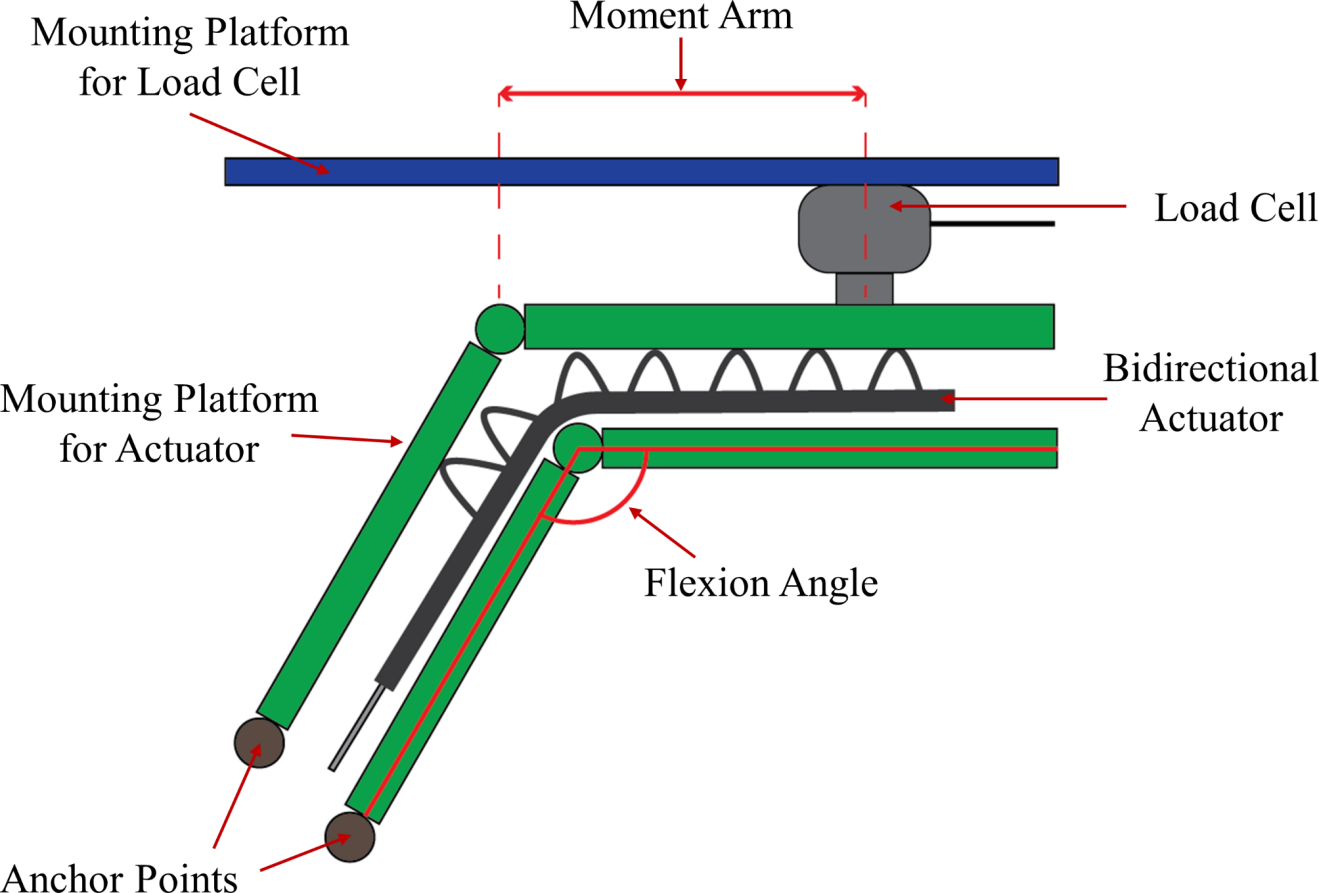
Test setup for Extension Torque measurements. The extension actuator is pressurized at 130 kPa, and static torque is measured at each flexion angle. Static torque is calculated as the product of the force measured by the load cell and the moment arm

The extension actuator is characterised by the amount of extension torque it can produce. The torque output was obtained using a customised torque testing rig, and a load cell sensor (Fig. 5). Using the torque testing rig, force exerted by the extending motion of the actuators is detected by the load cell as the mounting platform pushes against the load cell and is converted to static torque based on the distance from the load cell to the axis of rotation. The static torque of the extension actuator was measured from 0° to 90° in 10° intervals while the actuator was pressurized at 130 kPa.

### Study design

This study evaluated the efficacy of the SR Glove as an assistive device in enhancing hand function in chronic stroke patients with limited hand recovery in step-down care (IRB Approval: LH-19-014). The trials were conducted in a case series design, whereby each participant first completed the tasks in the unassisted condition and then repeated the tasks in the assisted condition, all in a single session.

**Fig. 6:**
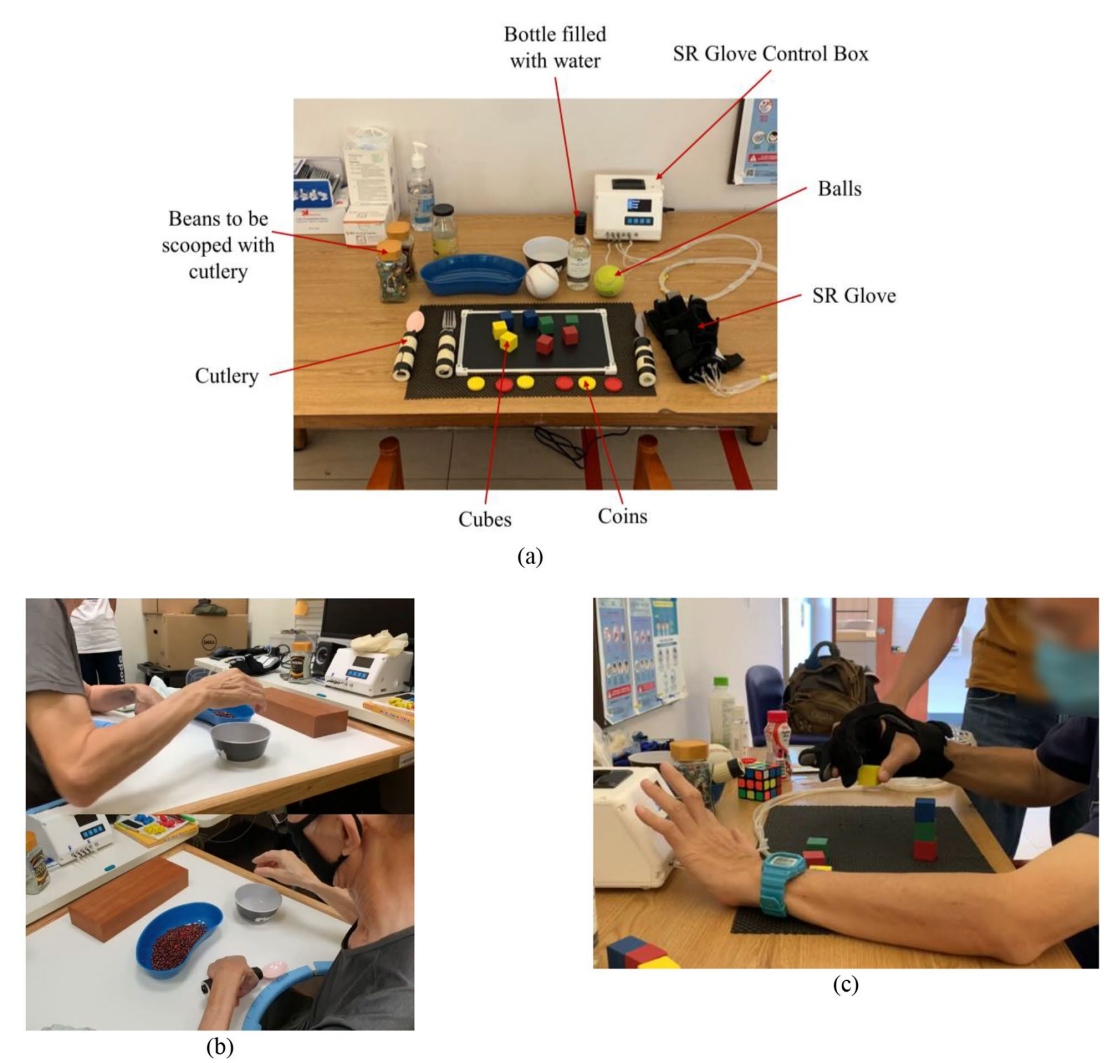
**(a)** Trial setup. **(b)** Gripping and manipulating utensils with the bare affected hand. **(c)** Performing functional tasks with the SR Glove on their paretic hand and operating the Robotic Glove Control Box with their contralateral hand

Unassisted hand function was first evaluated by assessing their performance in ADL tasks which can be split into three different categories. Firstly, grasping different sizes of blocks, bottles, and balls (Fig. 6a) (3 points per type of object, maximum of 9 points). Secondly, gripping and manipulating utensils (Fig. 6b) and pens (6 points maximum). Finally, pinching small cubes and coins (6 points maximum). The participants would then repeat the tasks in the assisted condition while wearing the SR Glove on their paretic hand (Fig. 6c).

Prior to beginning the tasks in the assisted condition, the participants were given a brief period of 10 min to familiarize themselves with controlling and using the SR Glove. During the tasks in the assisted condition, the participants were instructed to independently operate the SR Glove module by using their non-affected hand to press the buttons on the Robotic Glove Control Box, following the therapist’s instructions on which movements to choose and when to perform them. Additionally, the participants could also use their non-affected hands to transfer the objects to their affected hands in the case where they were unable to reach over to the objects if their affected arms were too weak. However, cooperation from the unaffected hand was not permitted for finger extension and flexion in the affected hand (i.e. using the unaffected hand to force open affected hand’s fingers to grip object).

The trials were held at local rehabilitation centers. The participants were recruited among regular patients in the rehabilitation centers, under referral by their therapists. Every participant underwent a proper procedure of recruitment, eligibility screening, and consent taking prior to the trial and had to agree with the procedures of the trial and data collection for research purposes. Participants’ demographics and research-related data were collected after successful enrolment such as gender, age, date of birth, date of diagnosis, type of stroke, duration of the stroke, and affected side.

After being successfully enrolled, the participants underwent pre-trial assessments to evaluate their grip strength by squeezing a hand dynamometer. After completing the hand functional tasks under both conditions, participants underwent a post-trial grip strength test. Lastly, a usability questionnaire (Appendix item A1) was given to patients to evaluate their level of confidence and reliance on support in ADLs.

### Participants and eligibility criteria

A total of eight chronic stroke patients participated in the study (Table 1). The enrolled patients were from two local rehabilitation centers (both centers are operated by Handicaps Welfare Association, Singapore). The inclusion criteria included patients suffering from chronic stroke, either ischemic or hemorrhagic; from the ages of 20 to 90 years regardless of gender and race; unilateral upper limb impairment; ability to comprehend and follow instructions; ability to maintain upright sitting for at least 30 min.

**Table 1:** Demographics of the Chronic Stroke Patients Recruited in the Study

Age	70 ± 8.28
Gender	Male = 5 Female = 3
Stroke Type	Ischemic = 5 Hemorrhagic = 3
Location	Cortex = 2 (e.g., occipital lobe, ACA) Subcortex = 7 (e.g., thalamus, basal ganglia, pons)
Affected Side	Left = 5 Right = 3
Duration (years)	5.41 ± 6.95
Grip Strength	8.29 ± 10.72
*Based on the voluntary movement, grip strength, and duration of the stroke, participants were classified into high and low functional groups.	

We excluded patients with recurrent stroke, unstable medical conditions, severe depression or active psychiatric disorder, epilepsy or seizure, severe spasticity (Modified Ashworth Scale > 2), contracture and deformity, poor skin conditions, and cognitive impairment. In addition, pregnant women were excluded from this study. All the participants were able to make decisions by themselves and give their consent to this trial.

### Outcome measures

#### Hand functional tasks

The hand functional tasks adapted for this trial by the study team were based on recognized and widely used hand assessments for stroke patients, such as Fugl-Meyer Assessment-Upper Extremity (FMA-UE), Action Research Arm Test (ARAT), and Arm Motor Ability Test (AMAT). These hand functional tasks allowed for observation and assessment of the participant’s abilities to perform ADLs with and without assistance from the SR Glove with a focus on how well the participants could extend their fingers to place the objects in their palms and let go of the object after the manipulation (“Hand Opening”) and how well they could flex their fingers to grip and manipulate the objects (“Hand Closing”). The assessments were measured on the affected hand and each task for the different trial items were scored from 0 to 3 for “Hand Opening” and “Hand Closing” separately. A zero score would be given for no voluntary movement. A one would be given for slight voluntary movement and inability to sustain the movement. A two would be given for obvious voluntary movement and the ability to sustain the movement for a longer time but lacks the functionality to complete the given task. And a three would be given for obvious voluntary movement, ability to sustain the movements and to complete the functional tasks.

#### Grip strength

The maximum bare-hand grip strength of the participants was measured by using a standard Jamar Hydraulic Hand Dynamometer. Patients were asked to position the studied hand in 90 degrees of elbow flexion next to the body and squeeze the hand dynamometer three times before the trial and three times after the trial, and force measurements were averaged.

#### Usability questionnaire (level of confidence in performing ADLs and reliance on support for ADLs)

After completing the trial, a questionnaire designed by the study team was given to the participants for their feedback on the experience using the SR Glove, their level of confidence and their reliance on support from caretakers in performing ADLs while being unassisted or assisted by the SR Glove.

The questionnaire had the participants indicate their confidence in performing the tasks based on the group of objects (bottle, block, pen, etc. – 5 points per object, 35 points maximum), as well as their reliance on support while performing ADLs (1 for highest reliance, 5 for lowest reliance) with and without the assistance from the SR Glove.

### Data collection and statistical analysis

All trials were video recorded for rating post-test. The outcome measures and participant responses were recorded and paired (unassisted versus assisted and pre-trial versus post-trial). The responses were coded, entered, and analyzed by using Statistical Package for Social Sciences (SPSS) version 25. Descriptive results such as the mean and the standard deviation were calculated. Given the small sample size, a nonparametric statistic test, the Wilcoxon Signed Ranks test, was applied to observe the differences in results between the two conditions (unassisted versus assisted by SR Glove) among the patients. A significance level of p-value ≤ 0.05 was used in the analyses.

## Results

### SR glove actuator characterization

#### Blocked tip force measurement

**Fig. 7:**
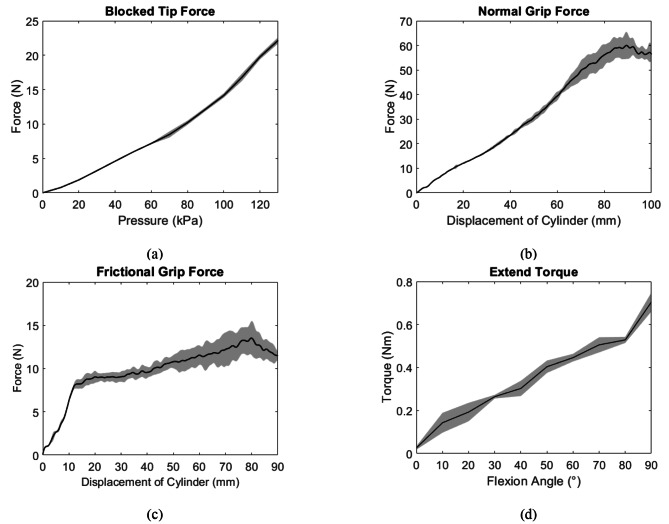
**(a)** Blocked tip force generated by the flexion actuators. **(b)** Normal grip force applied by the flexion actuators upon pressurization to 130 kPa. **(c)** Frictional grip force applied by the flexion actuators upon pressurization to 130 kPa. **(d)** Extension torque generated by a single extension actuator upon pressurization to 130 kPa at different flexion angles

The force produced by the SR Glove Actuators increased with greater pressure. The actuators generated a maximum tip force of 22.2 N at 130 kPa (Fig. 7a), which is also the operating pressure of the actuators. Compared to the previous iteration of a fully fabric Soft Robotic Glove [11], these actuators are able to produce a greater maximum tip force at their operating pressure.

#### Grip force measurements

The grip force measurements (normal grip force and frictional grip force) showed that the SR Glove Actuators generated 60.01 ± 5.50 N of normal grip force and 13.53 ± 1.93 N of frictional grip force using a cylinder with a diameter of 80 mm (Fig. 7b and c). The frictional grip force provided by the flexion actuators is sufficient to lift most objects of daily living, which typically do not weigh more than 1.5 kg [21].

#### Extension torque

Stroke patients with hand impairments typically have increased muscle tone in the finger flexors on their affected hand, leading to an unintentionally clenched hand. It is therefore essential that the SR Glove Actuator can produce enough extension torque to extend the users’ fingers to grasp objects successfully.

A single extension actuator was found to be able to generate a maximum of 0.70Nm at 90° (Fig. 7d). Assuming all extension actuators generate the same amount of torque, the maximum total extension torque achieved by four actuators would be 2.8Nm at 90°. Based on a previous study, the total finger flexion torque of stroke patients with spastic finger flexors is estimated to be within 0.5–4 Nm [22]. Therefore, the results suggest that the SR Glove can counteract a total flexion torque of up to 2.8Nm and assist in finger extension, depending on the severity of the participant’s spasticity.

### Trial with chronic stroke patients

#### Hand function – finger extension and finger flexion

**Fig. 8:**
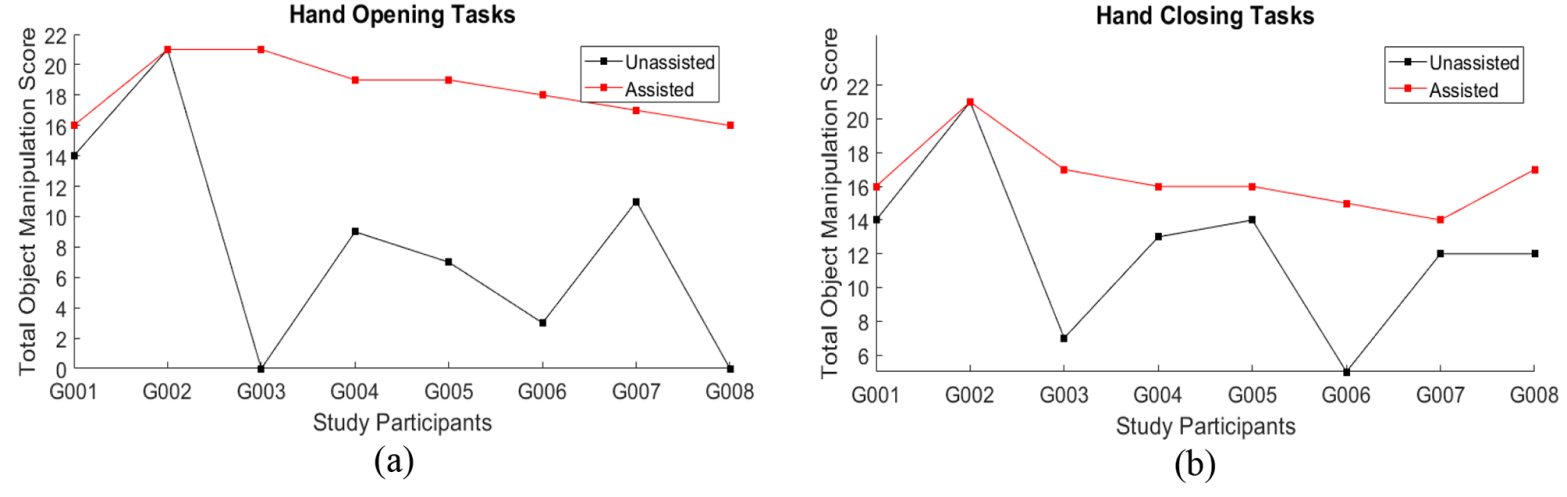
Individual participant scores in unassisted and assisted conditions for **(a)** Hand Opening tasks and **(b)** Hand Closing tasks

**Fig. 9:**
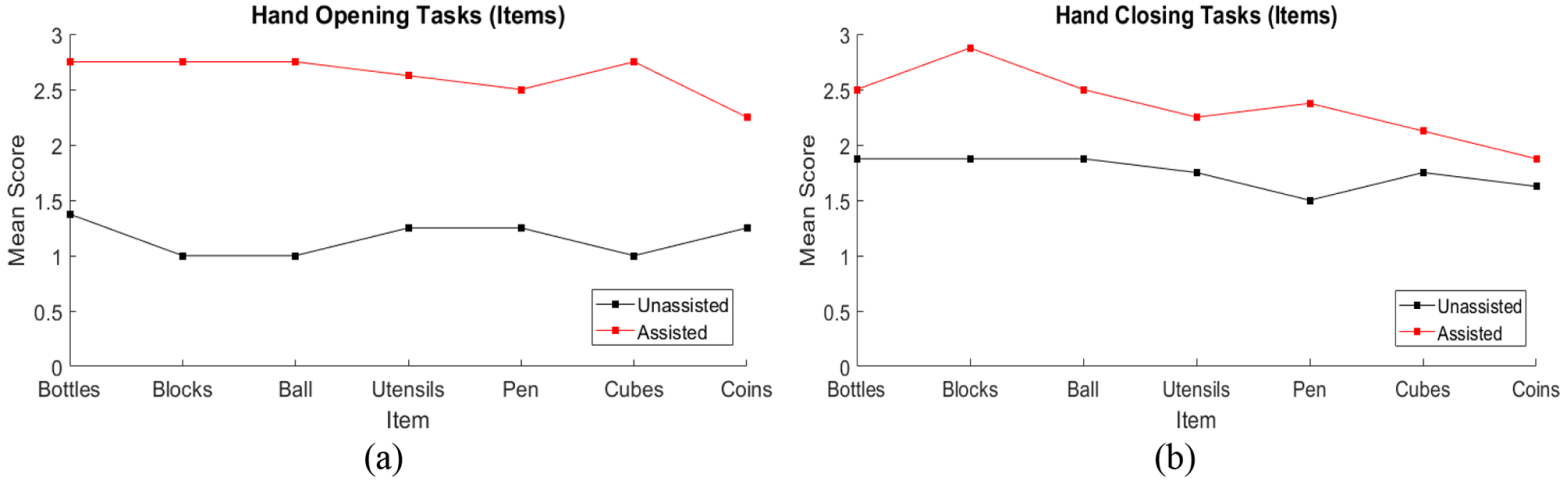
Mean participant scores, separated by the trial item, in unassisted and assisted conditions for **(a)** Hand Opening tasks and **(b)** Hand Closing tasks. Bottles, blocks and the ball represent the palmar grasp, utensils and pen represent the tripod pinch and cubes and coins represent the two-finger pinch

**Fig. 10:**
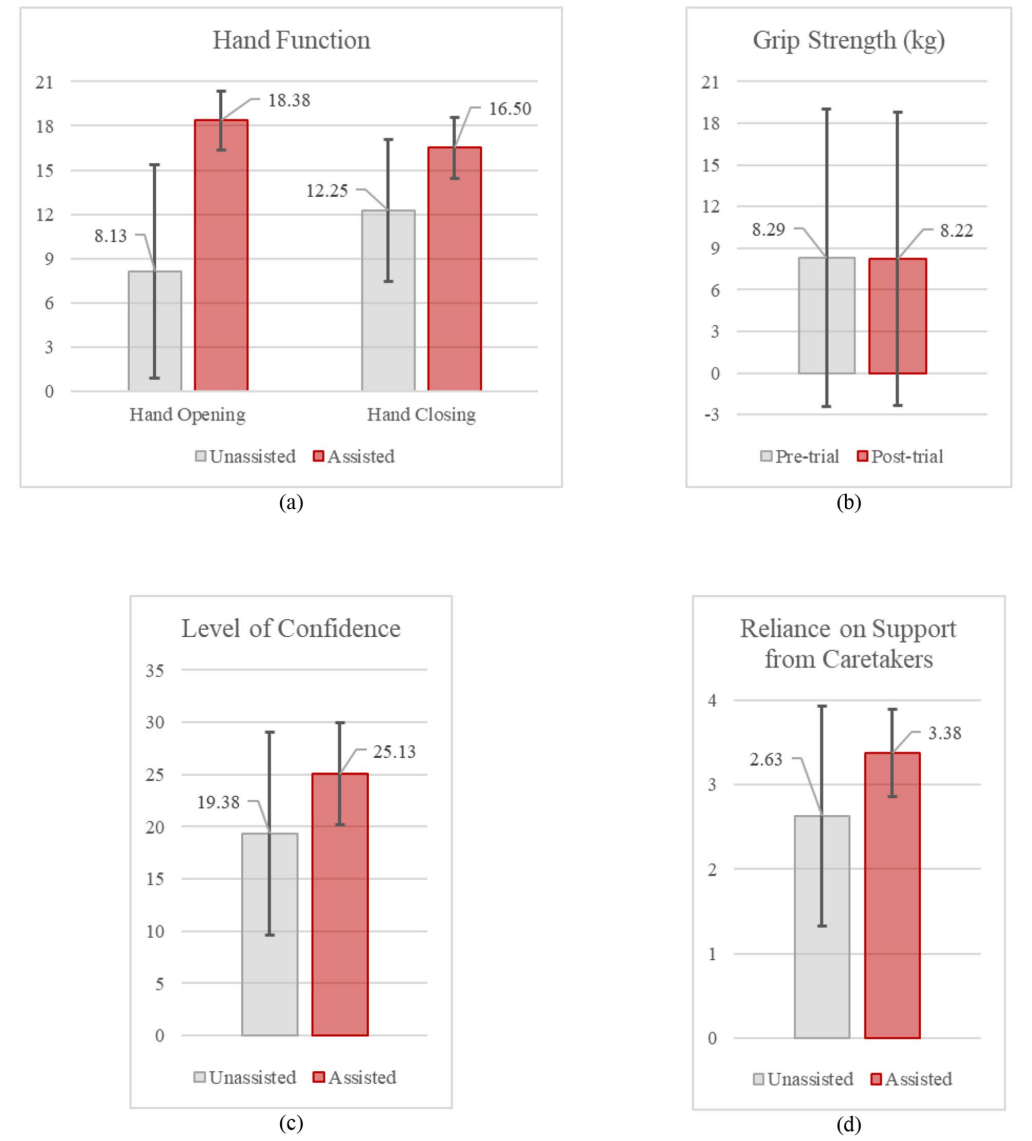
**(a)** Mean hand function scores across all study participants in the unassisted and assisted conditions, separated into tasks focusing on hand opening and hand closing **(b)** Mean grip strength across all study participants before commencing the trial and after the trial session. **(c)** Mean scores of the level of confidence to perform ADLs from the usability questionnaire done by the study participants, 5 points per object, 35 points maximum. Higher mean score in the assisted condition means the participants have a higher mean level of confidence while being assisted by the SR Glove. **(d)** Mean scores of the reliance of support from caretakers from the usability questionnaire done by the study participants (1 for highest reliance needed, 5 for lowest reliance needed). Higher mean score in the assisted condition means the participants feel they rely on support from others less when using the SR Glove

Individual participant scores in the hand function tasks, separated into tasks focused on hand opening and hand closing, are illustrated in Fig. 8a and b to provide insight into the score differences for each participant between unassisted and assisted conditions. The mean scores separated by the trial item are illustrated in Fig. 9. Mean results across all test participants for the hand opening and closing tasks in unassisted and assisted conditions are also presented in Fig. 10a; Table 2.

**Table 2:** Outcome Measures

Subject	Affected Side		Hand Opening		Hand Closing		Difference (Assisted – Unassisted)		Grip Strength (kg)		
			Unassisted	Assisted	Unassisted	Assisted	Open	Closed	Pre	Post	Difference
G001	L		14	16	14	16	2	2	25.50	24.92	-0.58
G002	R		21	21	21	21	0	0	25.00	24.90	-0.1
G003	L		0	21	7	17	21	10	2	3.27	1.27
G004	R		9	19	13	16	10	3	4.50	7.76	3.26
G005	R		7	19	14	16	12	2	2.33	1.93	-0.37
G006	L		3	18	5	15	15	10	0	0	0
G007	L		11	17	12	14	6	2	7.00	3.00	-4
G008	L		0	16	12	17	16	5	0	0	0
	Mean ± SD (total)		8.13 ± 7.26	18.38 ± 2.00	12.25 ± 4.83	16.50 ± 2.07	10.25 ± 7.23	4.25 ± 3.81	8.29 ± 10.72	8.22 ± 10.58	-0.07 ± 2.02

Hand Functional Tasks: the maximum score is 21.

Abbreviation: L: Left; R: Right; SD: Standard Deviation;

The mean score difference between the unassisted and assisted conditions improved significantly across the group of eight participants. The mean score for Open Hand Tasks in the assisted condition (18.38 ± 2.00) showed an improvement of more than double of the unassisted condition (8.13 ± 7.26). This shows a significant improvement in finger extension function when participants used the SR Glove (Wilcoxon signed-rank test, Z = -2.366, *p* = 0.018).

The mean score difference for the Closed Hand Tasks was similar. The mean unassisted score was found to be 12.25 ± 4.83 while the mean score for the assisted condition was 16.50 ± 2.07. This was also a significant increase in finger flexion function when participants used the SR Glove (Wilcoxon signed-rank test, Z = -2.388, *p* = 0.017).

#### Participant grip strength

The mean grip strength did not exhibit a statistically significant change post-trial (*p* = 0.753, Table 3; Fig. 10b). As the trials were conducted in a single session, an increase in grip strength, which would require an extended training timeline, was not expected. Furthermore, a decrease in grip strength was observed in some participants when comparing post to pre-trial, which could be due to fatigue after performing the functional tasks.

**Table 3:** Wilcoxon Signed Ranks Test Statistics for the Outcome Measures

	Hand Opening: Unassisted vs. Assisted	Hand Closing: Unassisted vs. Assisted	Grip Strength: Pre vs. Post
Z	-2.366	-2.388	− 0.314
Asymp. Sig. (2-tailed)	0.018*	0.017*	0.753
*Statistically significant, < 0.05.			

#### Usability questionnaire

The questionnaire done by the participants after completing the trial reflected their level of confidence and reliance on support for ADLs with and without the SR Glove (Fig. 10c and d; Table 4).

**Table 4:** Usability Questionnaire

Subject	Affected Side	Level of Confidence			Reliance on Support		
		Unassisted	Assisted	Difference	Unassisted	Assisted	Difference
G001	L	20	21	1	4	3	-1
G002	R	30	30	0	4	4	0
G003	L	7	20	13	1	3	2
G004	R	18	20	2	2	4	2
G005	R	28	28	0	3	3	0
G006	L	7	22	15	1	3	2
G007	L	31	32	1	4	3	-1
G008	L	14	28	14	2	4	2
	Mean ± SD (total)	19.38 ± 9.71	25.13 ± 4.88	5.75 ± 6.88	2.63 ± 1.30	3.38 ± 0.52	0.75 ± 1.39

Usability Questionnaire: the maximum score of Level of Confidence is 35; the maximum score of Reliance on Support is 5.

Abbreviation: L: Left; R: Right; SD: Standard Deviation;

Out of a maximum of 35 points, the participants rated their confidence in performing the ADL tasks in the unassisted condition with a mean score of 19.38, and a mean score of 25.13 while using the SR Glove. This increase in confidence was significant (Wilcoxon signed-rank test, Z = -2.207, *p* = 0.027) (Table 5).

**Table 5:** Wilcoxon Signed Ranks Test Statistics for the Questionnaire

	Level of Confidence: Unassisted vs. Assisted	Reliance on Support: Unassisted vs. Assisted
Z	-2.207	-1.622
Asymp. Sig. (2-tailed)	0.027*	0.105
*Statistically significant, < 0.05.		

Additionally, participants felt they required less support by caretakers while using the SR Glove, with a mean score of 3.38 in the assisted condition and a mean score of 2.63 in the unassisted condition (Fig. 10d), though this was not statistically significant.

## Discussion

The viability of a fabric-based soft robotic glove in assisting chronic stroke participants with hand impairments to perform ADLs was investigated in this study. The study team adapted a series of hand functional tasks (FMA-UE, ARAT and AMAT) to assess the hand manipulation of the participants in unassisted and assisted (using SR Glove) conditions, with a focus on finger extension and flexion, as well as object manipulation. Compared to the unassisted condition, the improved scores and reduction in variability when they were assisted by the SR Glove suggest that the glove is capable of enhancing the hand function of participants with impaired unaided performance to a more functional level. These results are consistent with previous studies [23–25]. Moreover, many studies on robotics in rehabilitation present improvements in motor functions and cortical excitability in stroke patients, most likely relating to neuroplasticity [26–28].

In this study, we found that individuals with low unassisted scores (G003 and G006) experienced great benefits from using the SR Glove, with great improvements to their hand functional scores. In contrast, individuals with higher unassisted scores (G001, G002) did not experience notable benefits from the SR Glove. We believe this may be due to the ceiling effect when assessing patients with higher hand functions who generally present better motor control and less overall spasticity. This outcome suggests the SR Glove might be more beneficial for patients who suffer from severe to moderate impairment and experience decreased hand strength, an inability to initiate movement, and have spastic hands [29, 30]. In addition, we also observed that the relatively large standard deviation from the unassisted condition was reduced in the assisted condition, suggesting the SR Glove provided consistent assistance across all participants. This result is similar to a study done by Cappello et al. that also observed a decrease in the variability of participants’ performance while wearing the soft robotic glove [31].

As is consistent among chronic stroke patients with muscle spasticity, our participants presented unintentionally clenched fists on their affected sides. To address this, the SR Glove Actuators are bidirectional and can assist the user in extending as well as flexing their fingers. Extension torque tests show the SR Glove can generate a peak torque of 2.8Nm. The participants demonstrated a lower unassisted mean score in Open Hand Tasks as compared to Closed Hand Tasks (Table 2), which suggests a greater difficulty for the participants to perform reliable finger extension than flexion. Assistance with the SR Glove provided a significant improvement to their Open Hand Tasks scores. G003 was unable to perform any Open Hand tasks in the unassisted condition as they could not perform finger extension without assistance. With the assistance of the SR Glove, G003 could easily extend their fingers to then grasp onto objects or release them. Reducing spasticity with robot-assisted training is also supported by a study conducted by Gandolfi et al., which suggests that it is as effective as conventional methods for spasticity reduction when combined with Botulinum toxin in chronic stroke patients with upper limb spasticity [32].

Besides the hand functional outcomes, the usability questionnaire done by the participants post-trial indicated that the participants have a higher level of confidence in performing ADLs while being assisted with the SR Glove than without. This may suggest that the participants viewed the SR Glove as an effective assistive device. Additionally, there were no responses with a decrease in confidence while using the SR Glove. Results from the questionnaire also indicated the participants felt they had a lower reliance on support from caretakers when using the SR Glove.

This study assessed the participants’ hand functions as well as their experience using the SR Glove Module. The participants operated the SR Glove themselves during the trial, using their non-affected hand to control the buttons on the Control Box. The intuitive design of the SR Glove module allowed them to familiarize themselves with operating it and perform the ADL tasks independently and successfully in a single session. This could have been a factor as to why the participants felt they could rely less on support from caretakers while performing ADLs with the SR Glove. Similarly, Li et al. conducted a mixed methods survey on the experiences of using rehabilitative robots and reported that stroke patients enjoyed using rehabilitative robots in therapy sessions. This was due to the rehabilitative robots providing greater variety in therapy choices, increasing the amount or intensity of treatment, and making the therapy accessible and convenient [33]. As a result, patients have a greater motivation to practice and this increases their level of confidence in performing ADL tasks, especially when used at home [34]. Given these advantages of rehabilitative robots, the whole system of the SR Glove is designed to be less bulky. The Control Box is made to be compact (16.4 cm x 10.5 cm x 14.9 cm) (Fig. 1b) and lightweight, which makes it easy for transportation as well as ideal for home use. Given that users can independently operate the SR Glove as demonstrated by the participants of this study, it is potentially feasible for the SR Glove to be used as an assistive device at home.

Studies have also suggested that a contributing factor to the plateau seen in poststroke recovery after 6 months is a neuromuscular adaptation to a standardized outpatient regimen of exercise [35]. To address this, introducing novel rehabilitation protocols and mass practice has shown that considerable motor improvement is still possible in the chronic stage. Using the SR Glove in therapy sessions could potentially be the novel rehabilitation protocol that chronic stroke patients in rehabilitation centers use to experience hand motor improvements in their chronic stage.

Table 6 shows a comparison of the features and capabilities of the SR Glove with other state-of-the-art hand exoskeletons driven by soft robotic actuators. As the SR Glove was designed with the aim to provide chronic stroke patients with an assistive device to be used independently at home, the focal point was minimizing the weight of the whole setup while ensuring the SR Glove can still deliver powerful assistance, all while being intuitive enough for the user to put on and control themselves. When compared to similar pneumatic hand exoskeletons, the SR Glove setup is relatively light (the glove presented by Zhou et al. [36] and Correia et al. [37] did not specify the weight of the control box), while providing the greatest max grip force. The cable-driven hand exoskeletons [13, 38] are lighter and more compliant than the SR Glove, but the tradeoff is a lower grip force and less fingers being actuated. It is good to note that all the papers [13, 31, 36–38] presenting the hand exoskeletons in the comparison table saw improved hand functions in their respective tests with patients with hand mobility impairments (Stroke and Spinal Cord Injury).

**Table 6:** Comparison to Soft Robotic Gloves from Relevant Studies

Glove Name / Paper	Material	Actuation	Weight	Fingers Actuated	Max Grip Force (N)	Independent Control
SR Glove	Fabric	Pneumatic - Bidirectional	Glove: 150.8 g Control Box: 1.36 kg	4 Fingers	60	Yes – Buttons on control box
Zhou et al., 2019 (36), Correia et al., 2020 (37)	Fabric	Pneumatic – Bidirectional	Glove: 149 g Control Box: N/A*	4 Fingers + Thumb	37	Yes – Buttons on the glove for other hand to press
Cappello et al., 2018 (31)	Fabric	Pneumatic – Bidirectional	Glove: 77 g Control box: 5 kg	4 Fingers + Thumb	15	No – Controlled by Study Researcher
Thimabut et al., 2022 (38)	Textile	Cable-driven – Flexion only	Glove: 42 g Control Box: 475 g	2 Fingers (Index and Middle)	28	Yes – Hand control switch
Kang et al., 2018 (13)	Silicone	Cable-driven – Bidirectional	Glove: 104 g Control Box: 1.14 kg	2 Fingers (Index and Middle)	10.6	Yes – Single button
*Authors did not provide weight of the control box.						

**Figures for “A Bidirectional Fabric-Based Soft Robotic Glove for Hand Function Assistance in Patients with Chronic Stroke”**

In addition, the SR Glove can be adapted to suit clinical environments. For example, the SR Glove can be used as a continuous passive motion device, simply extending and flexing the affected fingers to reduce spasticity in affected fingers. Alternative methods of control, such as electromyography (EMG), electroencephalography (EEG), and inertial measurement units (IMUs), could also be explored in the future to further enhance the operation of the SR Glove by the users. Therefore, future studies will aim to investigate the efficacy of the SR Glove in rehabilitative treatments.

A key limitation of this study was the small sample size. We recognized that variances in characteristics, such as stroke type and time elapsed since onset of stroke, might impact the consistency of the overall results from the study. As we conducted this study amid the COVID-19 pandemic, we had to follow strict restrictions on physical contact, greatly limiting the number of participants we could recruit. Nevertheless, we managed to ascertain the importance for home-based assistance in chronic stroke patients with hand motor impairments.

Future research will also need to investigate the durability and long-term usability of the glove while performing intensive testing sessions. Adjustments to the design of the SR Glove could improve the overall performance and user comfort while using the glove, such as adding silicone anti-slip material to the fingertips to aid gripping and incorporating a more suitable, thinner material on the palmar side of the glove to improve the precision of pinching and grasping objects. The study participants also provided feedback that they felt the actuation of the SR Glove was too slow, which affected the overall speed that they performed the ADL tasks in the trial. This led to them feeling less confident in their reliance on support from caretakers. Faster actuation, perhaps by means of a more powerful electronic air pump coupled with a proportional integral derivative (PID) controller, should therefore be implemented to this main concern. In addition, functional neuroimaging will provide a better understanding of the effect of robotic rehabilitation [26–28] and could be considered for future studies. Finally, adding sensors in the SR Glove will be essential in providing biofeedback to both patients and therapists; this will improve the training effect and efficacy [39, 40].

## Conclusion

This study provided insight into the efficacy of a fully fabric-based bidirectional soft robotic glove in improving basic hand functions in chronic stroke patients. Assessing the participants based on widely used hand assessments for stroke patients, assistance with the SR Glove showed significant improvements in finger flexion and extension when performing ADLs. Additionally, a usability questionnaire given to the participants showed that the participants had significantly greater confidence in performing the ADL tasks while using the SR Glove than without. These results, coupled with the SR Glove’s portability and intuitive operation, show that the SR Glove can be used both in rehabilitation center settings as well as in home environments as an assistive device to enhance hand function in chronic stroke patients with hand paresis.

### Electronic supplementary material

Below is the link to the electronic supplementary material.

Supplementary Material 1

## Abbreviations

ADL: Activities of daily living
SR: Soft robotics
FMA-UE: Fugl-Meyer Assessment-Upper Extremity
ARAT: Action Research Arm Test
AMAT: Arm Motor Ability Test
