# Supplementary material for: A bidirectional fabric-based soft robotic glove for hand function assistance in patients with chronic stroke
Source: J Neuroeng Rehabil. 2023 Sep 21;20:120. doi: 10.1186/s12984-023-01250-4 (PMC10512630; doi:10.1186/s12984-023-01250-4)

**Appendix for “A Bidirectional Fabric-Based Soft Robotic Glove for Hand Function Assistance in Patients with Chronic Stroke”**

**Appendix item A1: Questionnaire given to study participants after the trial.**


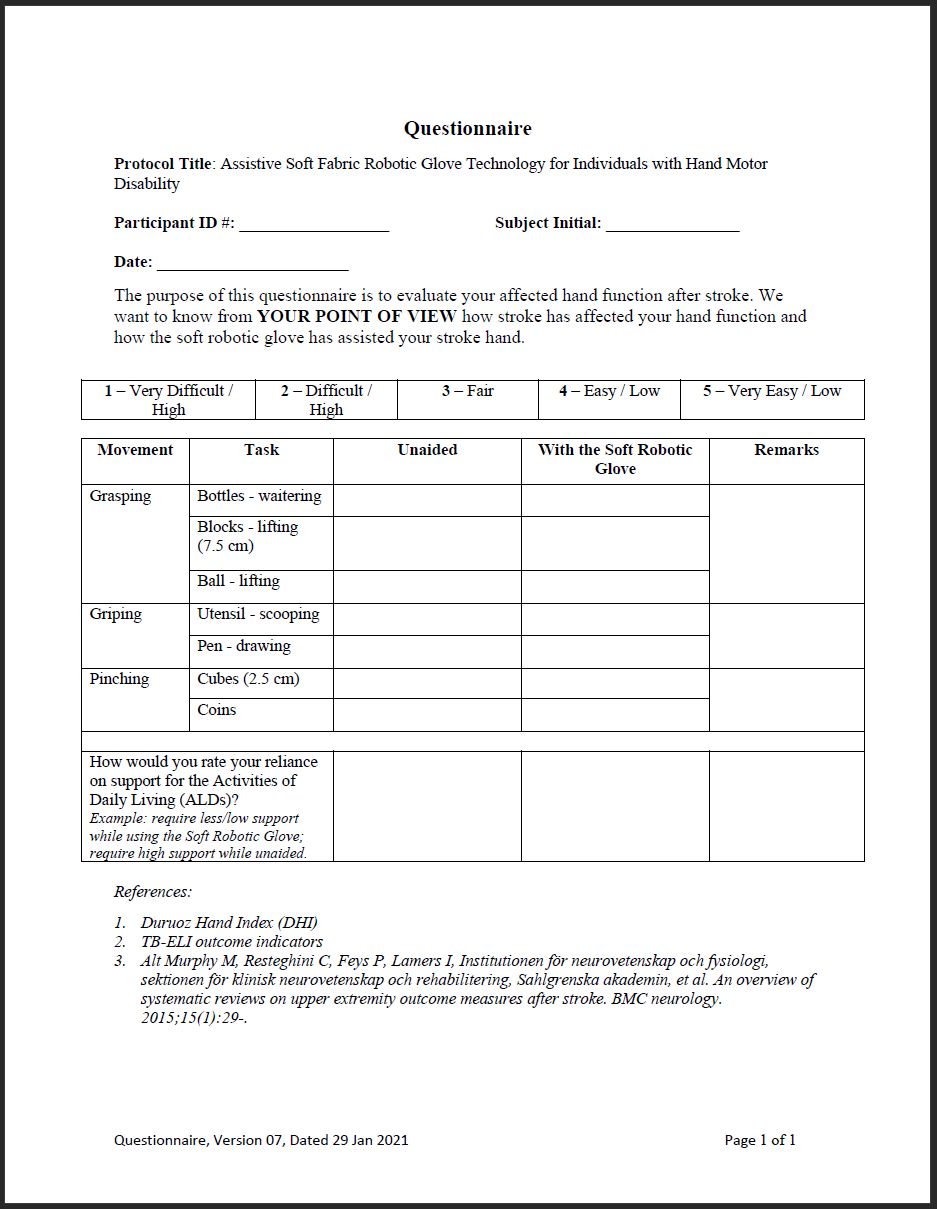

Supplement: Supplementary file 1 — Supplementary Material 1 [file 12984_2023_1250_MOESM1_ESM.docx]
